# Study on the relationship between functional characteristics and environmental factors in karst plant communities

**DOI:** 10.1002/ece3.9335

**Published:** 2022-09-20

**Authors:** Yang Wang, Limin Zhang, Jin Chen, Ling Feng, Fangbing Li, Lifei Yu

**Affiliations:** ^1^ Key Laboratory of Plant Resource Conservation and Germplasm Innovation in Mountainous Region (Ministry of Education), College of Life Sciences/Institute of Agro‐bioengineering Guizhou University Guiyang China; ^2^ Institute of Mountain Resources of Guizhou Academy of Sciences Guiyang China

**Keywords:** ecological strategy, environmental factors, functional traits, karst, vegetation recovery

## Abstract

Environmental factors drive changes in plant functional traits, which in turn promote community recovery. The environmental conditions of the community are different at different recovery stages. Changing environmental factors may drive the changes in plant functional traits at the community level and affect species adaptation. We studied plant communities in five different recovery stages (herb, grass and shrub, shrub, tree and shrub, and tree) in the karst plateau of Zhenning, Guizhou (The vegetation in the study area has undergone a gradual natural recovery process after the forests were deforested in 1958–1960). We studied functional traits and their links to environmental factors. The main results include the following. (1) Over time, plant height, leaf dry matter content, leaf nitrogen content and leaf phosphorus content increased significantly in the tree stage, while leaf thickness and specific leaf area decreased significantly in the tree stage. (2) Soil organic carbon, soil N content, soil P content, soil C:P and soil C:K showed an increasing trend, and were significantly higher in tree stage than in other stages. Soil potassium content fluctuated and soil bulk density decreased gradually, reaching the lowest value in the tree stage, but the difference was not significant. (3) During the restoration process, the functional characteristics changed from a combination of plant communities with high specific leaf area and low dry matter content with a short plant height to plant communities with low specific leaf area and high dry matter content with a tall plant height. (4) As recovery proceeded, the study area gradually changed from a soil nutrient‐poor environment to a nutrient‐rich environment. Overall, the environmental factors vary greatly during the recovery of plant communities in karst areas. The plant community shifts from an aggressive (resource acquisition) to a conservative (environmental barrenness resistance) ecological strategy. The soil phosphorus content and soil C:K are the main environmental factors affecting the changes in functional traits during the restoration of karst plant communities in Zhenning.

## Graphical abstract

Environmental factors drive changes in plant functional traits, which in turn promote community recovery. The environmental conditions of the community are different at different recovery stages. The changing environmental factors may drive the changes in plant functional traits at the community level and affect species adaptation. Using the plant communities at five different recovery stages (herb, grass and shrub, shrub, tree and shrub, and tree) in the karst plateau of Zhenning in Guizhou, we studied functional traits and their links to environmental factors. Overall, the environmental factors vary greatly during the recovery of plant communities in karst areas, plants shifted from an open ecological strategy to a conservative ecological strategy. The soil phosphorus content and C:K are the main factors influencing the change of functional traits during the recovery of karst plant communities in Zhenning.

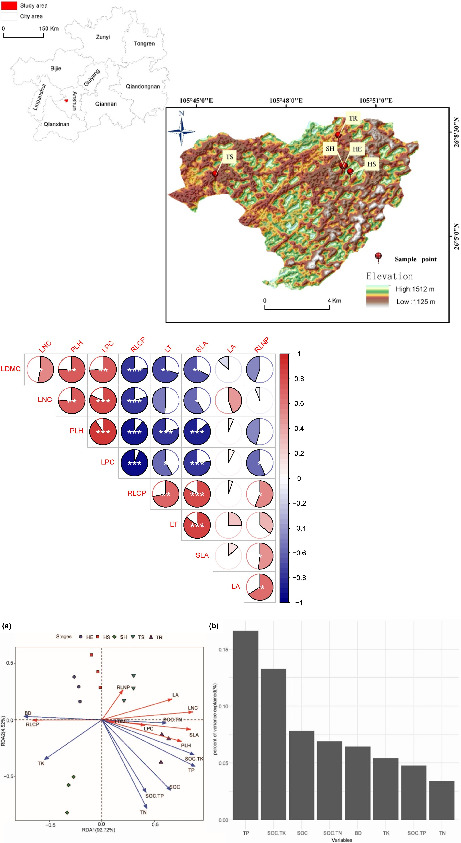

## 1 INTRODUCTION

Plant functional traits are expressions of plant function and morphology under different environmental conditions (Laughlin, 2014; Li et al., 2015; Poorter et al., 2018). They are often used to predict community structure and ecosystem functions (Diaz et al., 2007; Garnier et al., 2004), they influence plant survival, growth, and reproduction (Liu & Ma, 2015; Meng et al., 2007; Violle et al., 2007), and provide insights into ecological mechanisms such as biodiversity maintenance (Cadotte et al., 2015; Funk et al., 2016; Liu, Bai, et al., 2020b; Liu, Yu, et al., 2020a; Poorter et al., 2008). Leaf functional traits are closely related to the acquisition and utilization of plant resources and are sensitive to changes in environmental factors such as water, temperature, and light (Yao et al., 2014).

The study of functional traits can reveal the driving forces of community recovery following disturbance (Kahmen & Poschlod, 2010). At present, research on plant functional traits mainly explores the “static changes” of mature plants at a specific time (Liu et al., 2021). Soil factors affect the recovery of species and are considered to be strong influences on the functional traits of plants (Bu et al., 2013; Li et al., 2016). Environmental factors act as a “sieve” that determines which species or traits will be retained in a community. For example, specific leaf area decreases with increasing annual precipitation; leaf organic matter increases with increasing mean annual temperature (Shi et al., 2008); plant leaf N and P increase with elevation; and plant leaf N and organic carbon content increase with increasing soil water content (Liu & Ma, 2012). This variability in species' adaptations to the environment affects competitive advantages and leads to changes in community structure. Related studies have shown that as restoration proceeds, soil nutrients and water availability increase, there is a more complex community structure and reduced light resources in the lower forest canopy (Xu et al., 2015), and species that have lower resource acquisition capacity but higher resilience become more common (Castro et al., 2010; Muscarella et al., 2016). This can be reflected in trait shifts, such as decreasing specific leaf area and leaf nitrogen content (Bonal et al., 2007; Cortez et al., 2007), although this pattern can vary depending on the species and system (Craven et al., 2015; Reich et al., 2003).

The high rate of rock exposure, poor and discontinuous soils, and harsh ecological conditions have led karst forests to become fragile ecosystems (Yu et al., 2002a). The development of karst forests is influenced by the physiological ecology of plants, migration and population dynamics of animals, community succession, soil texture evolution and disturbance. Therefore, its distribution pattern is the result of multiple ecological processes. Multiple ecological processes are a general term for the flow and transformation of materials, energy and information within and between ecosystems in a region (or watershed) (Song et al., 2015). Previous studies have shown how plant composition is determined by habitat characteristics, such as the shallow karst soil layer, high soil water infiltration, and longer dry seasons, and thus plants may exhibit a combination of traits, such as low specific leaf area and high leaf dry matter content (Jiang et al., 2016; Liu & Ma, 2015; Wang et al., 2003; Xi et al., 2011). Studies on functional features in karst regions tend to concentrate on species‐level comparisons and less frequently take recovery sequences into account. In this study, we examined natural forests at different recovery stages in the Zhenning Karst plateau area to see how plant functional traits vary and link the variation to environmental changes. We focus on the following two questions: (1) What are the patterns of plant functional traits and ecological strategies of karst plant communities at different recovery stages? (2) What are the relationships between plant functional traits and environmental factors?

## 2 MATERIALS AND METHODS

### 2.1 Study area

**FIGURE 1:**
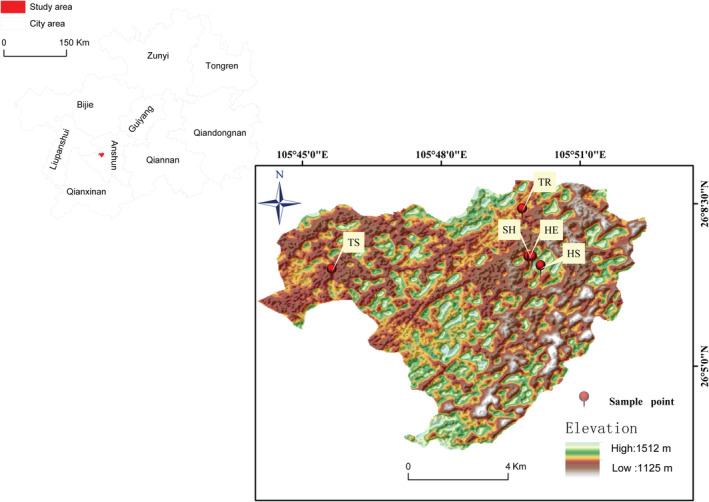
Location and sampling points of the study area. HE, herbage recovery stage; HS, herbage and shrub recovery stage; SH, shrub recovery stage; TR, tree recovery stage; TS, tree and shrub recovery stage.

The study area was a typical karst plateau in central Guizhou, which is located in Zhenning County, Anshun City, China (Figure 1). Zhenning County is located at 105°35′–106°01′E and 25°25′–26°11′N. The topography of the area is high in the north and low in the south, and the slope varies greatly (altitude of 447–2177 m). The region has a subtropical humid monsoon climate, with an annual average temperature of 16.2°C, the coldest month (January) with an average temperature of 6.5°C, and the hottest month (July) with an average temperature of 23.7°C. The annual frost‐free period is 297–345 days, the annual sunshine duration is 1142 h, and the annual average precipitation is 1277 mm. The parent rock of soil layer is limestone and the soil type is limestone. After deforestation in the study area from 1958 to 1960, the vegetation recovered gradually. In order to better analyze the relationship between plant functional characters and environmental factors in each restoration stage, five different restoration stages were selected as sample points. The five stages are herbage recovery stage, herbage and shrub recovery stage, shrub recovery stage, tree and shrub recovery stage and tree recovery stage. The dominant species in the herbaceous layer were *Imperata cylindrica*, *Carex capilliformis*, and *Miscanthus sinensis*; the dominant species in the shrub layer were *Pittosporum tobira*, *Pyracantha fortuneana*, *Rosa cymosa*, and *Coriaria nepalensis*; and the dominant species in the tree layer were *Platycarya strobilacea*, *Carpinus pubescens*, *Cyclobalanopsis argyroscopia*, and *Celtis sinensis*.

### 2.2 Sample plot, sample square setting, and community survey

This paper adopts a “space substitution for time” approach (Mueller‐Dombois & Ellenberg, 1974). First of all, it takes a long time for plants to grow. Secondly, when studying communities, it is difficult for us to have enough time to study the whole stage from grass to tree. Therefore, we can only select the existing plant communities at different stages for study, so that we can shorten the time of our plant survey as much as possible. In the study area, typical sample sites with similar elevation, slope, and slope directions were selected to set up sample plots. We sampled each plot only once. Sample plots were observed from May–June 2019 to identify sample plots; sample plots were sampled from September–November 2019. The sample plots were set up in the TR stage and TS stage with an area of 20 × 20 m; in the SH stage and HS stage with an area of 10 × 10 m; and in the HE stage with a sample plot of 5 × 5 m. Three replicate sample plots were set up in each stage. To verify the rationality of sample site selection, we performed *K*‐means clustering analysis on each sample site. We clustered the environmental factors of each restoration stage as variables and the sample sites of the restoration stage as cases. If the variability of TS clustering results is not significant, it indicates that our sample site selection is reasonable, and vice versa. The clustering results showed (Table 1) that three sample sites were selected in the TS stage, two of which were consistent with the SH stage and TR stage, with one sample site consistent with the HE and HS stage. No new category appeared in the TS stage, all of which could be clustered with other restoration stages. The reason for our inconsistent sample size at different stages is that we refer to previous studies. In the previous study, to obtain the minimum sample size for each stage, the researchers calculated a “species‐area curve” for this area to determine the minimum sample area for this area (Feng et al., 2011).

**TABLE 1:** Clustering results of sample sites in the restoration stages of the study area.

Case number	Sample	Clustering	Center distance
1	HE1	1	12.936
2	HE2	1	3.222
3	HE3	1	29.924
4	HS1	1	7.218
5	HS2	1	11.727
6	HS3	1	7.921
7	SH1	2	4.194
8	SH2	2	12.756
9	SH3	2	12.012
10	TS1	2	10.476
11	TS2	1	15.904
12	TS3	2	17.001
13	TR1	2	29.147
14	TR2	2	12.941
15	TR3	2	25.632

*Note*: The numbers following these abbreviated letters represent duplicate sample sites. In the Clustering list, the same number represents the same category. In the Center Distance list, the value represents the distance from the *K*‐means clustering to the center point.

Abbreviations: HE, herbage recovery stage; HS, herbage and shrub recovery stage; SH, shrub recovery stage; TR, tree recovery stage; TS, tree and shrub recovery stage.

We divided each standard sample plot into different sampling squares, and each squares was surveyed for tree layer, shrub layer, and herb layer. In the TR stage and TS stage, we divided four tree layer sampling squares with an area of 10 × 10 m. In each of the tree sampling squares, we selected a shrub sampling squares with an area of 5 × 5 m, and in each shrub sample square with an area of 1 × 1 m. In the HS stage and SH stage, we divided four shrub layer sampling squares with an area of 5 × 5 m, and in each of these shrub layer sampling squares, we selected an herbaceous sampling squares with an area of 1 × 1 m. In the HE stage, we randomly selected 10 sampling squares with an area of 1 × 1 m. Therefore, we surveyed 24 tree layer sampling squares, 24 shrub layer sampling squares, and 24 herb layer sampling squares in the TR stage and TS stage; 24 shrub layer sampling squares and 24 herb layer sampling squares in the SH stage and TS stage; and 10 herb sampling squares in the HE stage; a total of 130 small sampling squares were surveyed.

### 2.3 Selection of plant functional traits and determination of soil factors

Then, we recorded tree species, plant height (PLH), diameter at breast height, crown width and height under branches; shrub species, plant height and ground diameter; herb species, plant number, average height and cover. Environmental factors including elevation and slope direction were measured. The basic information of the dominant species in the sample site is shown in Table 2. We photographed and recorded the characteristic traits of the species, such as leaves, flowers, and fruits, and then compared the botanical histories to find out the names of the species. For trees under 5 m, we can directly use a tape measure to measure the height of the plant. For trees above 5 m, a pole of known length is set up on the ground according to the sun's irradiation, and the shadow length of the tree and the shadow length of the pole are measured separately, and then obtained according to the proportional relationship (the height of the tree is equal to the height of the pole multiplied by the shadow length of the tree and then divided by the shadow length of the pole). The breast diameter of a plant is measured by measuring its chest circumference with a tape measure about 1.3m above the ground. The method of measuring the ground diameter is to measure the ground diameter of the plant with a tape measure of about 0.1 m from the ground. The width of the crown is measured by measuring the shaded parts of the plant's leaves and branches that are cast vertically on the ground. The height under the branch is measured with a ruler when the plant grows its first branch. The count of the number of plants in the sample plot is called the number of plants. Average height is a measure of the average plant height of herbaceous plants. Coverage is estimated as a percentage of the distribution area of herbaceous plants in the sample plot. In total, we surveyed and measured 146 plant species.

**TABLE 2:** Basic information of dominant species in the sample site.

Recovery stage	Community type	Coverage (%)	Height (m)	DBH (cm)	Ground diameter (cm)	Elevation (m)	Aspect
Herbage (HE)	*Imperata cylindrica*, *Miscanthus sinensis* Community	90	0.75 ± 0.18a	N/A	1.1 ± 0.35a	1296 ± 2.65a	W
Herbage and shrub (HS)	*Rosa cymose*, *Carex capilliformis* Community	95	1.23 ± 0.27ab	N/A	2.6 ± 0.25b	1330 ± 4.16c	W
Shrub (SH)	*Pittosporum tobira* Community	95	2.24 ± 0.52b	N/A	3.4 ± 0.66c	1343 ± 2.89d	W
Tree and shrub (TS)	*Platycarya strobilacea*, *Pyracantha fortuneana* Community	90	6.58 ± 1.75c	7.3 ± 2.38a	N/A	1318 ± 8.96b	WN
Tree (TR)	*Platycarya strobilacea, Cyclobalanopsis argyrotricha* Community	89	11.07 ± 2.58d	12.4 ± 3.67b	N/A	1290 ± 5.29a	WN

*Note*: These numbers are mean values ± SD; We used multiple comparisons to test the statistical significance, when *p* < .05, the statistical treatment was statistically significant. Significant differences are indicated between a, b, c, d.

Abbreviations: DBH, diameter at breast height (mainly includes trees); Ground diameter, diameter at breast height of plant (mainly includes herbs and shrubs); Height, average plant height; W, the west; WN, the northwest.

Ten functional traits were selected: plant height (PLH), leaf thickness (LT), leaf dry matter content (LDMC), leaf area (LA), specific leaf area (SLA), leaf nitrogen content (LNC), leaf phosphorus content (LPC), leaf N:P (RLNP), leaf organic carbon content (LCC), and leaf C:P (RLCP). The measurements followed the New Global Manual for Standardized Measurement of Plant Functional Traits (Pérezharguindeguy et al., 2013). The top three plants (dominant species) in terms of importance at each stage were selected as sampling plants, and the branches in the southeast and northwest directions of the canopy of the sampling plants were cut with high pruning shears. About 20 healthy and disease‐free leaves were picked from each branch, and mix the dominant species in a sample plot into one sample. Significance value of tree layer = (relative abundance + relative frequency + relative dominance based on diameter at breast height)/3; significance value of shrub and grass layer = (relative abundance + relative frequency + relative cover)/3. We used these two equations to determine the dominant species of trees, shrubs and herbs. The fresh weight of the leaves of each plant sample was weighed. The samples were baked at 60°C for 72 h until they reached a constant weight. The dry weight of the leaves was weighed. LDMC = dry weight of the leaves/fresh weight of the leaves.

**FIGURE 2:**
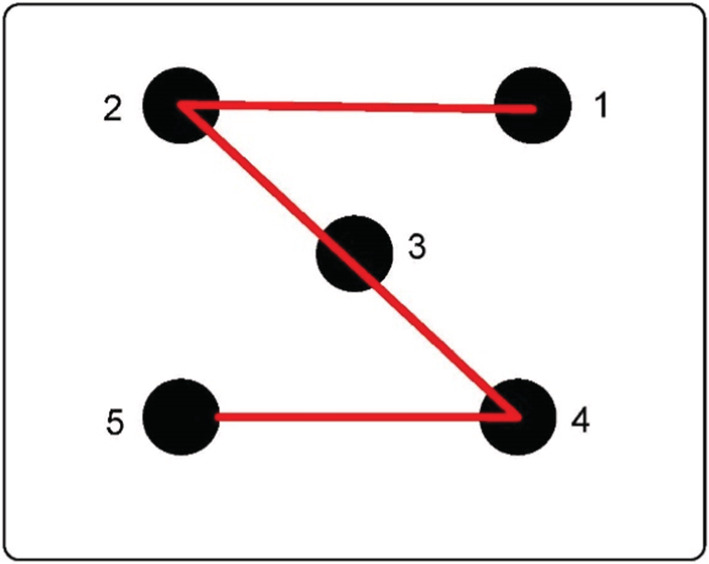
Schematic diagram of “S” type soil sampling. The black circles represent the soil sampling points, and the red lines represent the sampling routes. Because the red line is in the shape of “S”, it is called the “S” sampling method. 1–5 represent the order of soil sampling points.

The determination of blade thickness (LT) was made with electronic vernier calipers (Deli DL91150, Production country: China). Leaf length and leaf area (LA) were scanned and calculated by scanner combined with Photoshop software (Brand: HP; Model: HPScanJetN92120; Production country: China). SLA = Leaf area/leaf dry weight. Using the “S” sampling method (Long et al., 2004), the “S” sampling method is shown in Figure 2. Five soil samples were collected at depths of 0–30 cm (Liu, Bai, et al., 2020b; Liu, Yu, et al., 2020a). The steps for collecting soil samples are as follows: First, remove the debris from the soil surface, and then remove the plants and roots. Secondly, we use a ring knife to sample the soil at a depth of 0–30 cm, so as to get a soil sample. We store the bags with the plants and soil in a freezer in order not to destroy the samples. Soil samples were brought to the laboratory to measure soil bulk density (BD) and soil organic carbon (SOC), soil nitrogen content (TN), soil phosphorus content (TP), and soil potassium content (TK). The leaves were brought back to the laboratory for measurement of leaf carbon, nitrogen, and phosphorus content. The soil organic carbon and leaf organic carbon content were determined by the potassium dichromate oxidation‐external heating method (Bao, 2005). The leaf samples were decocted by H_2_SO_4_–H_2_O_2_ method, and the determination of leaf nitrogen content was sampled by indophenol blue colorimetric method and leaf phosphorus content was determined by molybdenum antimony anti‐colorimetric method (NY/T2017‐2011, 2011); soil nitrogen content was determined by Kjeldahl method and soil phosphorus content was determined by NaOH fusion‐molybdenum antimony anti‐colorimetric method (Bao, 2005; LY/T1228‐2015, 2015).

### 2.4 Data processing

Excel 2019 was used to organize trait data and soil factor data, and the FD package (Laliberté & Legendre, 2010) in R4.0.3 was used to calculate the community level functional trait values. One‐way analysis of variance and multiple comparisons (Tukey HSD) were used to analyze the environmental factors and functional traits of the communities at different recovery stages (Ryan, 2013). A Pearson correlation analysis was used to determine the correlation between functional traits (Wei & Viliam, 2021). The “Vegan” package was used to perform RDA analysis of environmental factors and plant functional traits (Lai et al., 2022). The “heir.part” package was used to perform the hierarchical partitioning analysis of environmental factors and functional traits (Ralph & Christopher, 2004). The statistical analysis and graphing were carried out in R4.0.3 (R Core Team, 2020).

## 3 RESULTS

### 3.1 Functional trait characteristics of plants in different recovery stages

**FIGURE 3:**
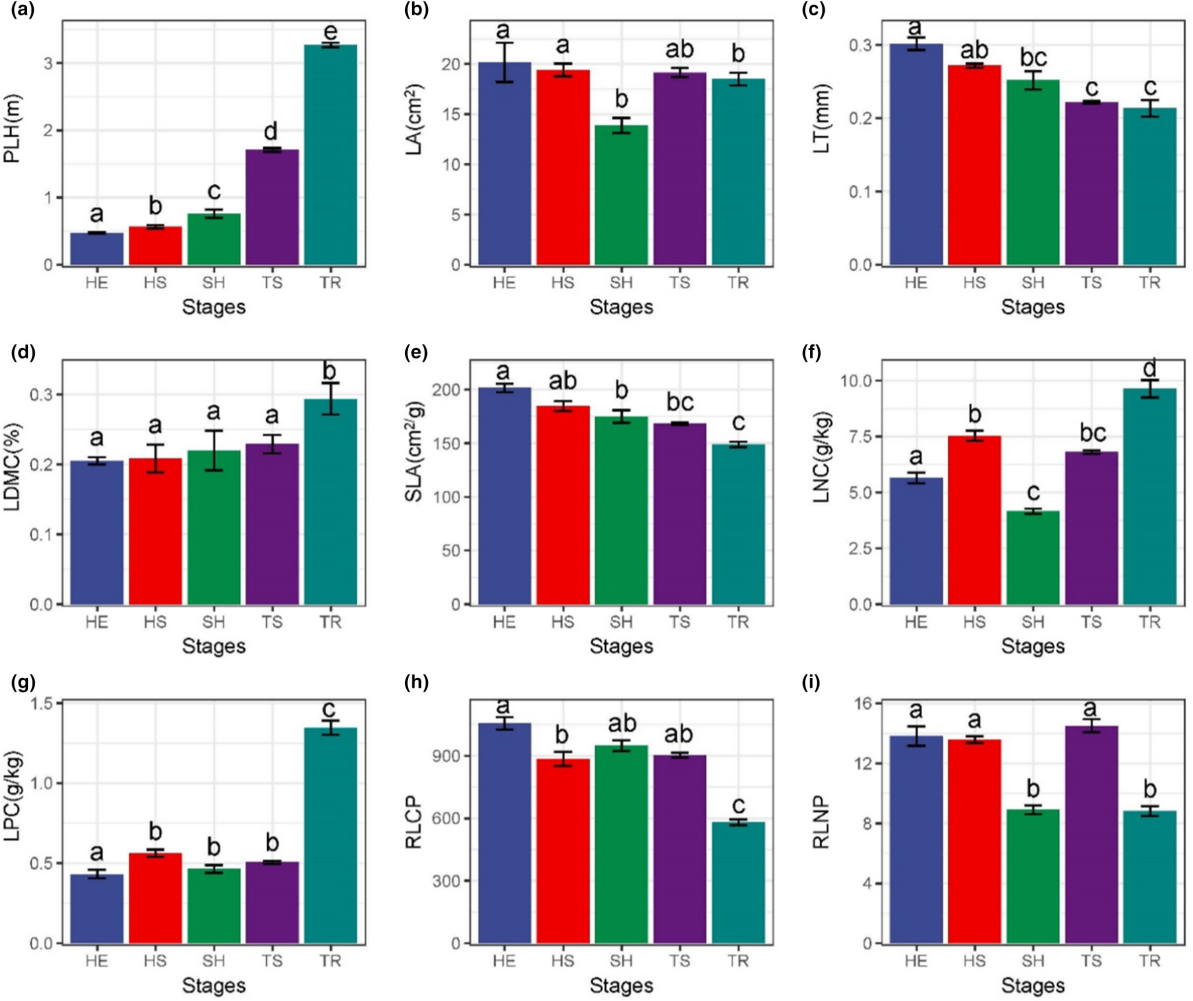
Change of plant functional properties at different recovery stages. In statistics, one‐way ANOVA is used for comparative analysis, when *p* < .05, it is statistically significant; Different lowercase letters between (a, b, c, d) represent significant differences. Bar graphs show mean (±95% confidence interval, CI); The error bar is the standard error (SE). Three individuals were measured per replicate, and three replicates were performed for each recovery stage. (a–i) The change characteristics of different plant functional traits with different recovery stages. The *Y*‐axis of each vignette represents the plant functional traits, and the *X*‐axis represents the recovery stage. The abbreviations of HE, HS, SH, TS and TR refer to Figure 1. LA, leaf area; LDMC, leaf dry matter content; LNC, leaf nitrogen content; LPC, leaf phosphorus content; LT, leaf thickness; PLH, plant height; RLCP, leaf C:P; RLNP, leaf N:P; SLA, specific leaf area.

As shown by the change of plant functional properties at different recovery stages (Figure 3). With the plant community recovered, PLH gradually increased and showed significant differences at each stage, with TR stage (3.35 m) significantly higher (*p* < .001) than TS stage (1.62 m) (Figure 3a); LNC showed an increasing trend, and the TR stage (9.64 g/kg) having significantly higher (*p* < .001) nitrogen content than the previous four stages, with HS stage (7.53 g/kg) being significantly higher (*p* < .001) than HE (5.65 g/kg) and SH (4.13 g/kg) stages, and the SH stage having the lowest nitrogen content (Figure 3f); LPC showed an increasing trend, and the TR stage (1.35 g/kg) was significantly higher than the rest of the stages (*p* < .001) (Figure 3g). LDMC in the TR stage (0.29) was significantly higher than in other stages (*p* = .03) (Figure 3d). LA showed a decreasing trend and was significantly higher in the HE (20.15 cm^2^) and HS (19.41 cm^2^) stages than in the SH (13.88 cm^2^) and TR (18.50 cm^2^) stages (*p* < .01) (Figure 3b). LT and SLA decreased gradually during recovery, and LT and SLA in HE stage were significantly higher than those in other stages, and LT (0.21 mm, *p* = .02), SLA (148.86 cm^2^/g, *p* < .001) in the TR stage was significantly lower than in the rest of the stages (*p* < .001) (Figure 3c,e); The RLCP gradually decreased and was significantly higher in the HE stage (1055.82) and lower in the TR stage (580.63) than in the rest of the stages (*p* < .001) (Figure 3h); the RLNP showed fluctuating changes, and was significantly higher in the HE stage (12.82), HS (13.57) and TS (14.51) stages than in the SH (8.90) and TR (8.82) stage (*p* < .001) (Figure 3i).

### 3.2 Characteristics of environmental factor changes in different recovery stages

**FIGURE 4:**
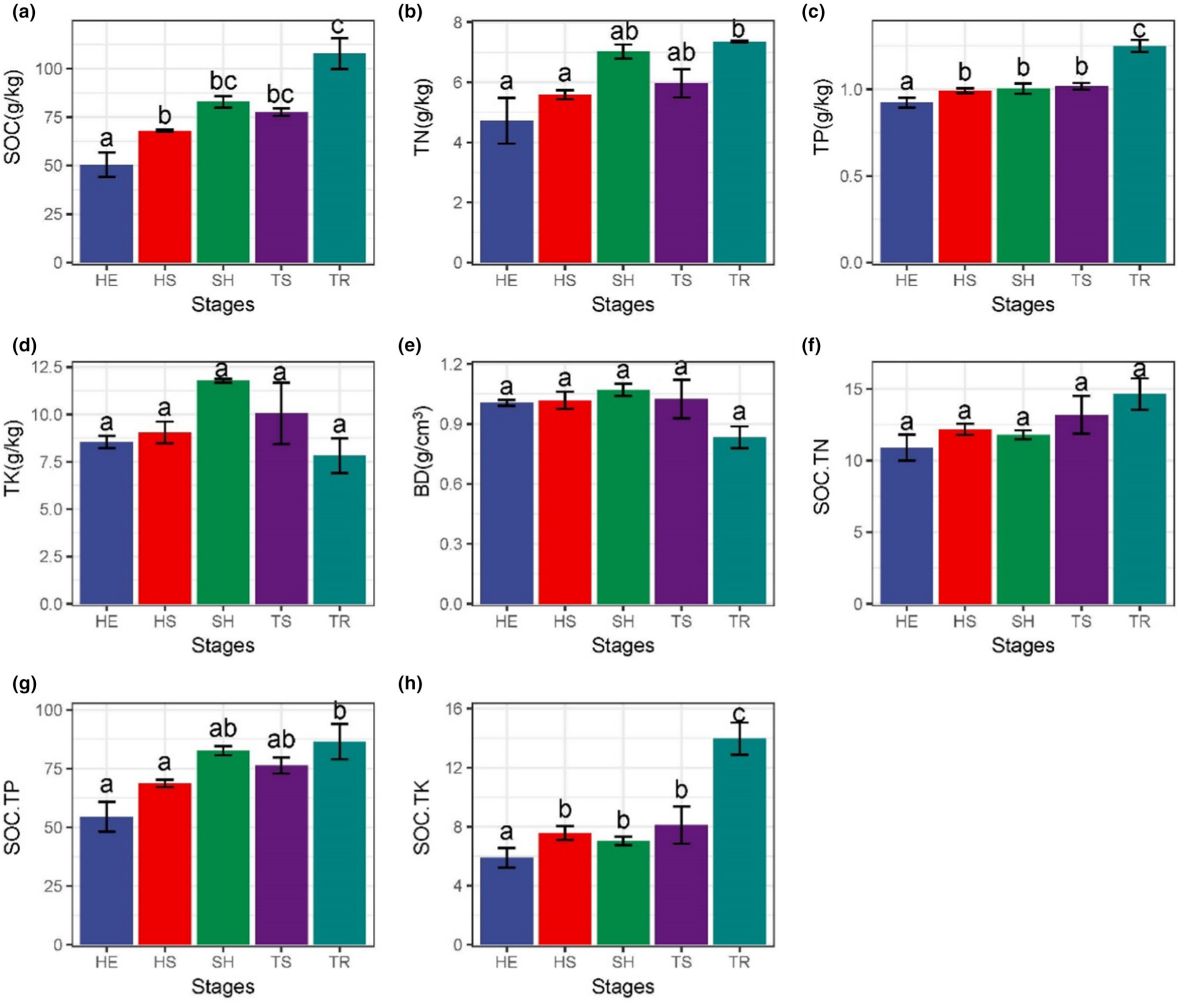
Change of environmental factors between different recovery stages. In statistics, one‐way ANOVA is used for comparative analysis, when *p* < .05, it is statistically significant; Different lowercase letters between (a, b, c) represent significant differences. The bar graphs show mean (±95% confidence interval, CI); The error bar is the standard error (SE). Three replicate experiments for each restoration phase of soil. (a–h) The change characteristics of different environmental factors with different recovery stages. The *Y*‐axis of each vignette represents the environmental factors and the *X*‐axis represents the recovery stage. The abbreviations of HE, HS, SH, TS and TR refer to Figure 1. BD, soil bulk density; SOC, soil organic carbon; SOC.TK, soil C:K; SOC.TN, soil C:N; SOC.TP, soil C:P; TK, soil potassium content; TN, soil nitrogen content; TP, soil phosphorus content.

As shown by the Change of environmental factors between different recovery stages (Figure 4). With the plant community recovered, SOC, TN, TP, SOC.TP, and SOC.TK showed an increasing trend, all showing significantly lower in the HE stage than in the rest of the stages. SOC (107.77 g/kg, *p* < .001) (Figure 4a), TN (7.35 g/kg, *p* < .001) (Figure 4b), and SOC.TP (86.52, *p* < .001) (Figure 4g) were significantly higher in the TR stage than in the rest of the stages. TP and SOC.TK did not differ significantly in the three intermediate stages (HS, SH, TS) and the tree stage was significantly higher than the remaining stages (*p* < .001) (Figure 4h). TK (*p* = .13), BD (*p* = .15), and SOC.TN (*p* = .23) in each stage were not significantly different (Figure 4d–f), TK was highest in SH stage (11.78 g/kg), BD was lowest in the TR stage (0.83 g/cm^3^), and SOC.TK was highest in the TR stage (13.97).

### 3.3 Correlation between functional traits

**FIGURE 5:**
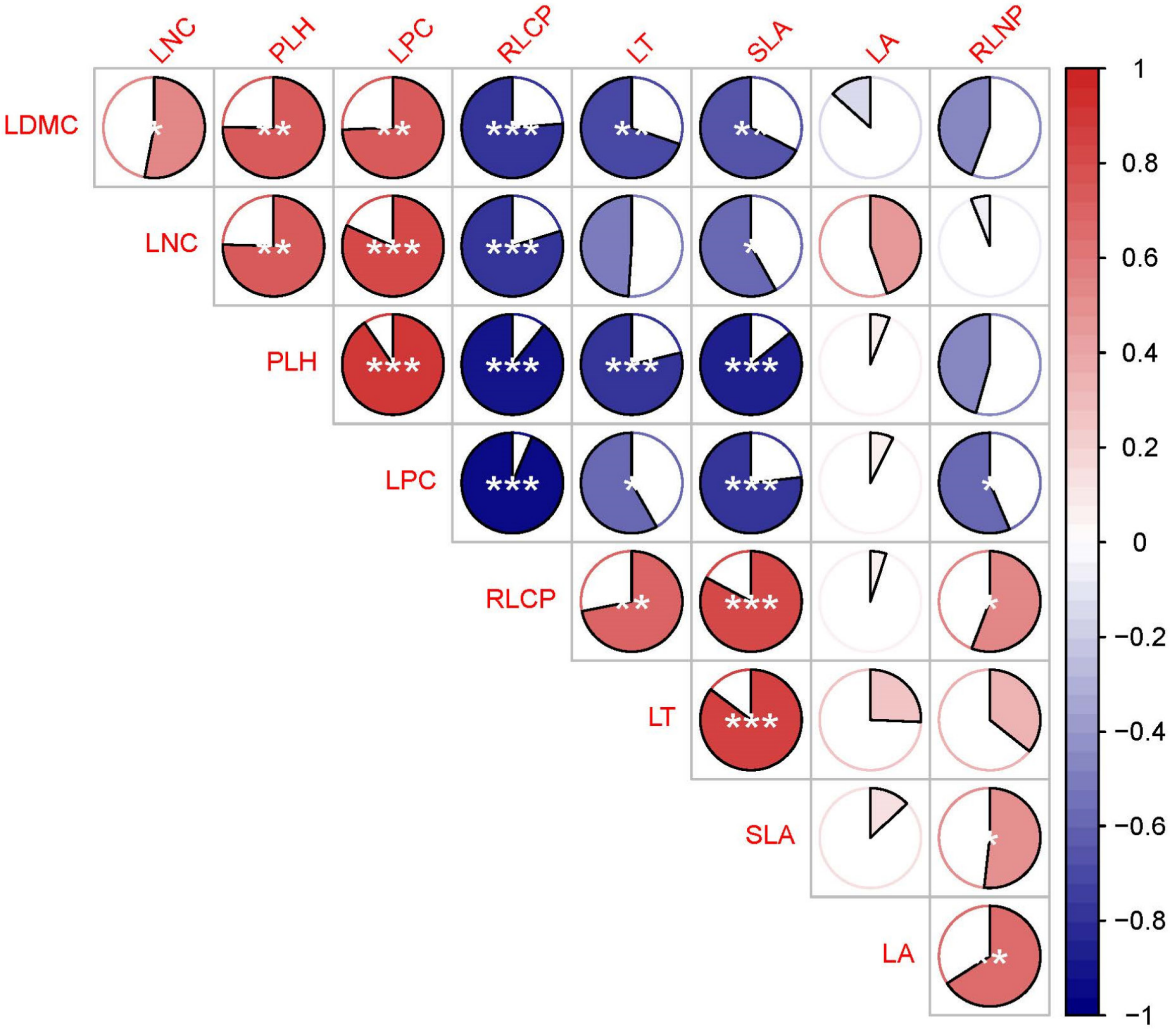
Correlation of functional characteristics of plant communities in the study area. ***p < .001; **p < .01; *p < .05. Red indicates positive correlation, and blue indicates negative correlation. The abbreviations of PLH, LT, LDMC, LA, SLA, LNC, LPC, RLNP and RLCP refer to Figure 3.

As shown by the correlation of functional characteristics of plant communities in the study area (Figure 5). With the plant community recovered, LDMC has a significant positive correlation with LNC, PLH, LPC, and a significant negative correlation with RLCP, LT, SLA; LNC had a significant positive correlation with PLH and LPC, and a significant negative correlation with RLCP and SLA; PLH had a significant positive correlation with LPC, and a significant negative correlation with RLCP, LT, and SLA; LPC is significantly negatively correlated with RLCP, LT, SLA, and RLNP. There was a significant positive correlation between RLCP and LT, SLA, and RLNP. There was a significant positive correlation between LT and SLA and a significant positive correlation between RLNP and both SLA and LA.

### 3.4 Effects of environmental factors on functional traits

**FIGURE 6:**
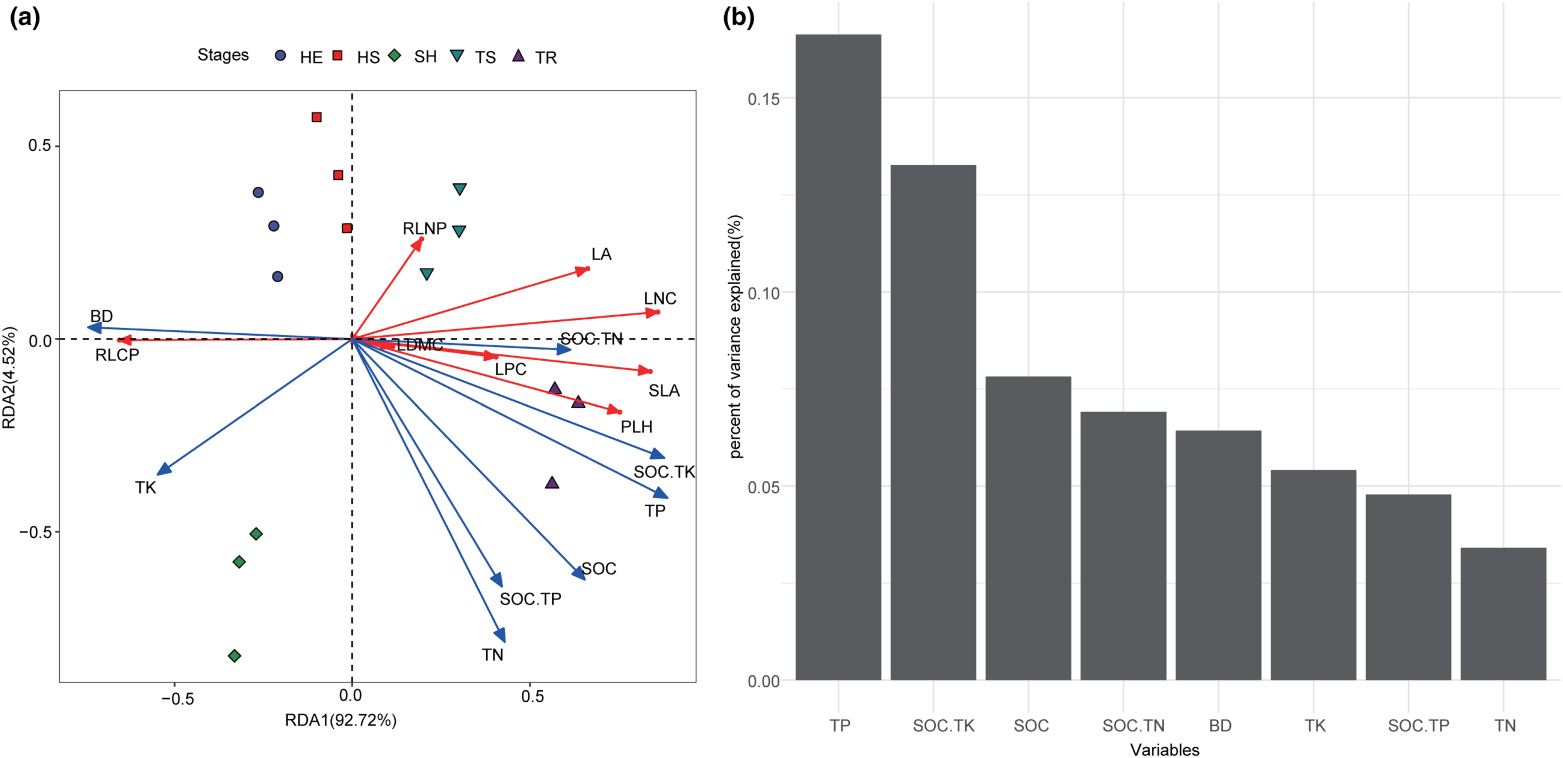
RDA sequencing diagram of plant functional traits and environmental factors. (a) The RDA ranking diagram of environmental factors and plant functional traits; (b) The hierarchical analysis of environmental factors on plant functional traits. Red lines are plant functional traits and blue lines are environmental factors. The abbreviations of HE, HS, SH, TS and TR refer to Figure 1. The abbreviations of PLH, LT, LDMC, LA, SLA, LNC, LPC, RLNP and RLCP refer to Figure 3. The abbreviations of SOC, TN, TP, TK, BD, SOC.TN, SOC.TP and SOC.TK refer to Figure 4.

As shown by the RDA analysis (Figure 6a), the overall percent of variance explained of environmental factors for plant functional traits in different recovery stages reached 92.72%, and the explanation rate of the first two axes of RDA reached 97.24%. TP, SOC.TK, SOC, SOC.TN, and BD were the main environmental factors affecting the changes in functional traits (Figure 6b). PLH and LPC were positively correlated with SOC.TN, SOC.TK, TP, SOC.TP, SOC and TN. LA, RLNP, and LNC were positively correlated with SOC.TN, SOC.TK, and TP, and had a negative correlation with TK. RLCP was positively correlated with TK and BD. The HE stage and HS stage were mainly affected by high soil BD and relatively low nutrients; the SH stage and TS stage were mainly affected by high soil BD and TK. The TR stage was mainly affected by relatively high soil SOC, TN, and TP.

**FIGURE 7:**
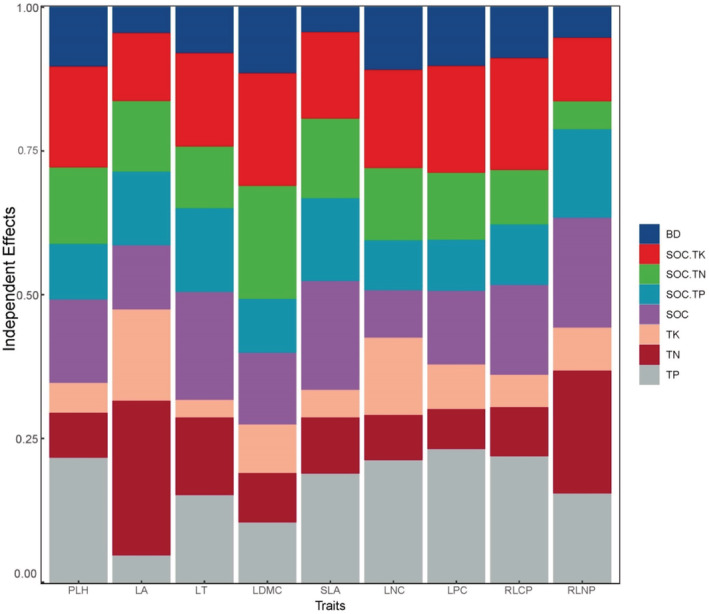
Functional traits are explained by environmental factors. The abbreviations of PLH, LT, LDMC, LA, SLA, LNC, LPC, RLNP and RLCP refer to Figure 3. The abbreviations of SOC, TN, TP, TK, BD, SOC.TN, SOC.TP and SOC.TK refer to Figure 4.

As shown by the functional traits are explained by environmental factors (Figure 7). PLH was mainly influenced by TP and SOC.TK; LA was mainly affected by TN and TK; LT was mainly influenced by SOC and TP; LDMC was mainly influenced by SOC.TK and SOC.TN; SLA was mainly influenced by TP and SOC. The influence factors of LNC, LPC and RLCP are similar, mainly affected by TP and SOCK. TK. LNC is also influenced by TK. RLNP was mainly influenced by TN, TP, and SOC.

## 4 DISCUSSION

### 4.1 Sensitivity of plant functional traits to environmental factors

Plant functional traits can reveal plant adaptation strategies and resource allocation strategies (Guittar et al., 2016). In this recovery study, PLH, LDMC, LNC, and LPC of plant community in the study area increased, LA, LT and SLA decreased gradually, and RLNP and RLCP had a tendency to decrease. SLA can reflect the plant's acquisition of light and water, and reflect the plant's ability to use resources (Cheng et al., 2019; Garnier et al., 2001; Pang et al., 2019; Wang et al., 2016). LDMC can reflect the degree of plant adaptation to environmental stress (Li et al., 2021). The combination of traits with low SLA and high LDMC indicated the stronger ability of plant communities to utilize environmental resources (Liu, Bai, et al., 2020b; Liu, Yu, et al., 2020a). The study area was a typical karst plateau, a rocky landscape with relatively scarce soil and water resources. Over time, plant leaves have improved water use efficiency via reducing transpiration. Plant communities enhanced their suitability for the environment by improving nutrient use and water conservation capacity, which is consistent with previous findings (Li et al., 2021; Liu et al., 2021; Zhang et al., 2019).

During the recovery process, PLH increased significantly, indicating that the competitiveness, productivity, and recovery ability of vegetation after disturbance gradually increased. LT gradually decreases, indicating that plant leaves have a strategy to store water in the early stage of recovery. Later, as LA decreased (Figure 3), the light penetration decreased and plants fully captured light resources by decreasing LT (Zhang, 2014). In this study, it was found that LNC and LPC increased at later stages which may be related to the nutrient content of the soil—these trends were consistent with the changes of soil TN and TP (Figure 6a). This further indicates that soil nutrients have a regulatory effect on leaf functional traits. The traits of the karst plant community changed through the various stages of restoration. The transformation process of plant traits is from high SLA and low LDMC traits to low SLA and high LDMC traits. At the beginning of the restoration stage, soil moisture and nutrients are low and the community has the ability to acquire resources quickly. We call this change an ecologically open strategy; at the later stage of the restoration stage, soil moisture and nutrients gradually increase, the community structure is complex, and the efficiency of the community in using water and nutrients is enhanced. We call this change an ecologically conservative strategy. The ecological strategy also changed from an active strategy to a conservative strategy, which was similar to the conclusions drawn by Garnier et al. (2004), Xi et al. (2011), and Wright et al. (2004).

Leaf N and P content is a direct reflection of plant nutrient supply and growth rate (Elser et al., 2003), and leaf N: P can be diagnosed as a limiting element during plant growth. A ratio less than 14 is limited by N, greater than 16 is limited by P, and between 14 and 16 is limited by both N and P (Koerselman & Meuleman, 1996). In this study, the N:P values of plant leaves in SH and TR stages were far less than 14, indicating that plant leaves in these two recovery stages were significantly limited by N. In addition, N:P of plant leaves in HE, HS and TS stages was about 14 (13.85, 13.28, 14.33), indicating that plant leaves in these three stages were restricted by N and P.

### 4.2 Correlations of plant functional traits to environmental factors at different recovery stages

The results of this study showed that TP, SOC.TK, SOC, SOC.TN, and BD were the main soil factors that influenced the changes in functional traits (Figure 6b). At the early stage of recovery (HE and HS stage), under low soil nutrients, the dominant species were *Imperata cylindrica*, *Miscanthus sinensis*, *Rosa cymosa*, *Carex capilliformis*, and other fast‐growing annual or biennial herbs and shrubs. This stage is mainly characterized by a combination of traits with high SLA and low LDMC, as well as a higher ability to acquire resources. The environmental factors in the middle recovery period (SH and TS stages) are mainly influenced by the TK, and the plant nutrient content is limited by the combination of N and P. It was a community with *Platycarya strobilacea* and *Cyclobalanopsis argyrotricha* as the dominant species with a conservative strategy and a combination of high PLH, LDMC, LNC, and LPC, and low SLA and LT. With the increase of soil P, plant leaf P content increased significantly and adapted to the strong light and poor soil environment by reducing water transpiration and increasing water use efficiency (Ackerly et al., 2002). Under the influence of environmental heterogeneity, there are great differences in leaf characters between species and within species (Jiang et al., 2016; Liu et al., 2015; Xi et al., 2011), but they generally show a combination of low specific SLA, LA and high LDMC. These trait combinations imply that plants tend to develop a combination of drought resistant traits to acclimate to habitat characteristics such as shallow karst soils layer and high soil water seepage (Li et al., 2021).

In general, soil bulk density can reflect soil water content. Soil water content can promote plant cell division and growth (Pang et al., 2019), and soil organic carbon can affect soil fertility and plant growth (Liu, 1995). The main soil impact factors in this study were TP > SOC.TK > SOC. It is speculated this is because the soil layer in karst areas is shallow and water and soil loss are high. There are often convergent or divergent ecological strategies among different communities, and functional traits will respond to the environment (Vile et al., 2006). The study area is a typical karst landscape development, with complex habitat structure, rich microhabitat composition, and obvious habitat heterogeneity. However, with the restoration of plant communities, the habitats in karst areas gradually transition from heterogeneity to homogeneity (Yu et al., 2002b). From the variance explained by environmental factors on functional traits (Figure 6), it can be seen that soil TP and SOC.TK had a greater influence on PLH, LNC, LPC, and RLCP of plants in the process of karst recovery. Combined with the results in Figure 4, it can be seen that in the early recovery stage, the environmental factors regulating plant functional traits were mainly soil BD; in the late recovery stage, it was showed that multiple environmental factors jointly regulated plant functional traits, especially in the TR stage showed more prominent performance. This also indicates that environmental factors have a stronger shaping effect on functional traits in the late recovery stage of karst plant communities. Therefore, with the process of change from heterogeneity to homogeneity in karst habitats, soil factors show a change from soil bulk density to comaintenance of multiple soil nutrient elements. To accommodate the change in soil factor, the functional traits also changed from the previous active type combination to the conservative type combination.

## 5 CONCLUSION

Based on the results of our study, the following conclusions were obtained.

1. In the process of natural recovery of karst plant communities, plant functional traits changed from an active ecological strategy to a conservative ecological strategy.
2. Environmental factors varied in different recovery stages of karst plant communities, and soil phosphorus content and soil C:K were the main influencing factors of functional trait changes in the study area.

## AUTHOR CONTRIBUTIONS

**Yang Wang:** Conceptualization (lead); data curation (lead); methodology (lead); visualization (lead); writing – original draft (lead); writing – review and editing (lead). **Limin Zhang:** Data curation (supporting); methodology (lead); writing – review and editing (supporting). **Jin Chen:** Data curation (supporting); methodology (supporting); writing – review and editing (supporting). **Ling Feng:** Data curation (supporting); methodology (lead). **Fangbing Li:** Methodology (supporting); writing – original draft (supporting). **Lifei Yu:** Conceptualization (equal); funding acquisition (equal); methodology (equal); project administration (equal); writing – original draft (lead); writing – review and editing (lead).

## FUNDING INFORMATION

This research was funded by the Construction Program of Biology First‐class Discipline in Guizhou (GNYL [2017]009), and the Project of National Key Research and Development Program of China (2016YFC0502604), and the Project of Promoted Innovation for Colleges and Universities of Guizhou Province (Qian Jiao He Collaborative Innovation [2014]01), and the Natural Science Research Project of Guizhou Provincial Department of Education (Qian Jiao He KY Zi [2018]170).

## CONFLICT OF INTEREST

The authors state that they have no conflicting interests.

### OPEN RESEARCH BADGES

All of our data (including survey data) is uploaded to Dryad. https://doi.org/10.5061/dryad.vx0k6djt6.

## Data Availability

All data are openly available in the public data repository Dryad; https://doi.org/10.5061/dryad.vx0k6djt6.

## References

[ece39335-bib-0001] Ackerly, D. , Knight, C. , Weiss, S. , Barton, K. , & Starmer, K. (2002). Leaf size, specific leaf area and microhabitat distribution of chaparral woody plants: Contrasting patterns in species level and community level analyses. Oecologia, 130, 449–457. 28547053 10.1007/s004420100805

[ece39335-bib-0002] Bao, S. D. (2005). Soil agrochemical analysis (3rd ed., pp. 45–52). China Agricultural Press.

[ece39335-bib-0003] Bonal, D. , Born, C. , Brechet, C. , Coste, S. , Marcon, E. , Roggy, J. , & Guehl, J. (2007). The recoveryal status of tropical rainforest tree species is associated with differences in leaf carbon isotope discrimination and functional traits. Annals of Forest Science, 64, 169–176.

[ece39335-bib-0004] Bu, W. S. , Zang, R. G. , Ding, Y. , Zhang, J. Y. , & Ruan, Y. Z. (2013). Ralationships between plant functional at the community level and environmental factors during recovery in a tropical lowland rainforest on Hainan Island, South China. Biodiversity Science, 21, 278–287.

[ece39335-bib-0005] Cadotte, M. W. , Arnillas, C. A. , Livingstone, S. W. , & Yasui, S. E. (2015). Predicting communities from functional traits. Trends in Ecology & Evolution, 30, 510–511. 26190136 10.1016/j.tree.2015.07.001

[ece39335-bib-0006] Castro, H. , Lehsten, V. , Lavorel, S. , & Freitas, H. (2010). Functional response traits in relation to land use change in the Montado. Agriculture, Ecosystem & Enviroment, 137, 183–191.

[ece39335-bib-0007] Cheng, W. , Yu, Y. H. , Xiong, K. N. , Zhang, Y. , Xu, M. , & Tan, D. J. (2019). Leaf functional traits of dominant species in karst plateau‐canyon areas. Guihaia, 39, 1039–1049.

[ece39335-bib-0008] Cortez, J. , Garnier, E. , Perez‐harguindeguy, N. , Debussche, M. , & Gillon, D. (2007). Plant traits, litter quality and decomposition in a Mediterranean old‐field recovery. Plant & Soil, 296, 19–34.

[ece39335-bib-0009] Craven, D. , Hall, J. S. , Berlyn, G. P. , Ashton, M. S. , & van Breugel, M. (2015). Changing gears during recovery: Shifting functional strategies in young tropical secondary forests. Oecologia, 179, 293–305. 25990298 10.1007/s00442-015-3339-x

[ece39335-bib-0010] Diaz, S. , Lavorel, S. , de Bello, F. , Quetier, F. , Grigulis, K. , & Robson, T. M. (2007). Incorporating plant functional diversity effects in ecosystem service assessments. Proceedings of the National Academy of Sciences of the United States of America, 104, 20684–20689. 18093933 10.1073/pnas.0704716104 PMC2410063

[ece39335-bib-0011] Elser, J. J. , Acharya, K. , Kyle, M. , Cotner, J. , Makino, W. , Markow, T. , Watts, T. , Hobbie, S. , Fagan, W. , Schade, J. , Hood, J. , & Sterner, R. W. (2003). Growth rate–stoichiometry couplings in diverse biota. Ecology Letters, 6, 936–943.

[ece39335-bib-0012] Feng, J. C. , Shi, S. , Zhao, C. J. , & Liu, Y. (2011). Ecological experiment. Minzu University of China Press.

[ece39335-bib-0013] Funk, J. L. , Standish, R. J. , Stock, W. D. , & Valladares, F. (2016). Plant functional traits of dominant native and invasive species in mediterranean‐climate ecosystems. Ecology, 97, 75–83. 27008777 10.1890/15-0974.1

[ece39335-bib-0014] Garnier, E. , Cortez, J. , Billes, G. , NavaS, M. L. , Roumet, C. , Debussche, M. , Laurent, G. , Blanchard, A. , Aubry, D. , Bellmann, A. , Neill, C. , & Toussaint, J. P. (2004). Plant functional markers capture ecosystem properties during secondary recovery. Ecology, 85, 2630–2637.

[ece39335-bib-0015] Garnier, E. , Laurent, G. , Bellmann, A. , Debain, S. , Berthelier, P. , Ducout, B. , Roumet, C. , & Navas, M. L. (2001). Consistency of species ranking based on functional leaf traits. The New Phytologist, 152, 69–83. 35974476 10.1046/j.0028-646x.2001.00239.x

[ece39335-bib-0016] Guittar, J. , Goldberg, D. , Klanderud, K. , Telford, R. J. , & Vandvik, V. (2016). Can trait patterns along gradients predict plant community responses to climate change? Ecology, 97, 2791–2801. 27859101 10.1002/ecy.1500

[ece39335-bib-0017] Jiang, Y. , Chen, X. , & Ma, J. (2016). Interspecific and intraspecific variation in functional traits of subtropical evergreen and deciduous broadleaved mixed forests in karst topography, Guilin, Southwest China. Tropical Conservation Science, 9, 1–9.

[ece39335-bib-0018] Kahmen, S. , & Poschlod, P. (2010). Plant functional trait responses to grassland recovery over 25 Years. Journal of Vegetation Science, 15, 21–32.

[ece39335-bib-0019] Koerselman, W. , & Meuleman, A. F. M. (1996). The vegetation N:P ratio a new tool to detect the nature of nutrient limitation. Journal of Applied Ecology, 33, 1441–1450.

[ece39335-bib-0020] Lai, J. S. , Zou, Y. , Zhang, J. L. , & Peres‐Neto, P. (2022). Generalizing hierarchical and variation partitioning in multiple regression and canonical analysis using the rdacca.hp R package. Methods in Ecology and Evolution, 13, 782–788. 10.1111/2041-210X.13800

[ece39335-bib-0021] Laliberté, E. , & Legendre, P. (2010). A distance‐based framework for measuring functional diversity from multiple traits. Ecology, 91, 299–305. 20380219 10.1890/08-2244.1

[ece39335-bib-0022] Laughlin, D. C. (2014). Applying trait‐based models to achieve functional targets for theory‐driven ecological recovery. Ecology Letters, 17, 771–784. 24766299 10.1111/ele.12288

[ece39335-bib-0023] Li, D. , Kang, S. R. L. , Zhao, M. Y. , Zhao, M. Y. , Zhang, Q. , Ren, H. J. , Ren, J. , Zhou, J. M. , Wang, Z. , Wu, R. J. , & Niu, J. M. (2016). Relationship between soil nutrients and plant functional traits in different degradation stages of *Leymus chinensis* steppe in Nei Mongolia. Journal of Plant Ecology, 40, 991–1002.

[ece39335-bib-0024] Li, F. , Li, J. , & Long, J. (2015). Effects of vegetation types on soil organic carbon and nitrogen in typical karst mountainous areas. Chinese Journal of Ecology, 34, 3374–3381.

[ece39335-bib-0025] Li, Y. J. , Zheng, J. M. , Wang, G. Z. , Zhou, J. X. , Liu, Y. G. , & Ha, W. X. (2021). A study of functional traits of natural secondary forests and their influencing factors in different recovery stages in karst areas: A case study of Dahei Mountain, Yunnan Province. Acta Geologica Sinica, 42, 397–406.

[ece39335-bib-0026] Liu, H. W. , Liu, W. D. , Wang, W. , Chai, J. , & Tao, J. P. (2015). Leaf traits and nutrient resorption of major woody species in the karst limestone area of Chongqing. Acta Ecologica Sinica, 35, 4071–4080.

[ece39335-bib-0027] Liu, N. , Yu, L. F. , Zhao, Q. , Wu, Y. N. , & Yan, L. B. (2020a). C:N:P stoichiometry of leaf‐litter‐soil continuum in secondary forests of the rocky desertification regions of the karst plateau. Chinese Journal of Applied & Environmental Biology, 26, 681–688.

[ece39335-bib-0028] Liu, R. H. , Bai, J. L. , Bao, H. , Nong, J. L. , Zhao, J. J. , Jiang, Y. , Liang, S. C. , & Li, Y. J. (2020b). Variation and correlation in functional traits of main woody plants in the Cyclobalanopsis glauca community in the karst hills of Guilin, southwest China. Journal of Plant Ecology, 44, 828–841.

[ece39335-bib-0030] Liu, X. J. , & Ma, K. P. (2015). Plant functional traits‐concepts, applications and future directions. Scientia Sinica Vitae, 45, 325–339.

[ece39335-bib-0031] Liu, X. X. , & Ma, J. Z. (2012). Response of plant functional characters and soil factors to slope direction in Gannan alpine meadow. Chinese Journal of Applied Ecology, 23, 3295–3300. 23479869

[ece39335-bib-0032] Liu, X. X. , Nan, X. N. , Zhang, G. J. , Li, B. W. , Xu, L. , Mu, R. L. , Li, L. , & Yu, R. G. (2021). Relationship between species diversity and functional diversity of plant communities on different slopes in alpine meadow. Acta Ecologica Sinica, 41, 1–11.

[ece39335-bib-0033] Liu, Y. F. (1995). A study on the carbon cycle in the Agro‐ecological system of China. Journal of Natural Resources, 10, 1–8.

[ece39335-bib-0034] Long, J. , Li, J. , & Jiang, X. R. (2004). Soil microbial activities in Maolan karst forest, Guizhou Province. Acta Pedologica Sinica, 41, 598–602 (In Chinese).

[ece39335-bib-0035] LY/T 1288‐2015 . (2015). Determination of nitrogen in forest soils. China Standard Publishing House.

[ece39335-bib-0036] Meng, T. T. , Ni, J. , & Wang, G. H. (2007). Plant functional traits, environmental and ecosystem functions. Journal of Plant Ecology, 52, 150–165.

[ece39335-bib-0037] Mueller‐Dombois, D. , & Ellenberg, H. (1974). Aims and methods of vegetation ecology (p. 547). John Wiley and Sons.

[ece39335-bib-0038] Muscarella, R. , Uriarte, M. , Aide, T. M. , & Erickson, D. L. (2016). Functional convergence and phylogenetic divergence during secondary recovery of subtropical wet forests in Puerto Rico. Journal of Vegetation Science, 27, 283–294.

[ece39335-bib-0039] NY/T 2017‐2011 . (2011). Determination of nitrogen, phosphorus and potassium in plants. China Standard Publishing House.

[ece39335-bib-0040] Pang, Z. Q. , Lu, W. L. , Jiang, L. S. , Jin, K. , & Qi, Z. (2019). Leaf traits of different growing plants in karst area of Shilin, China. Guihaia, 39, 1126–1138.

[ece39335-bib-0041] Pérezharguindeguy, N. , Díaz, S. , & Garnier, E. (2013). New handbook for standardised measurement of plant functional traits worldwide. Australian Journal of Botany, 61, 167–234.

[ece39335-bib-0042] Poorter, L. , Castilho, C. V. , Schietti, J. , Olivera, R. S. , & Costa, F. R. S. (2018). Can traits predict individual growth performance? A test in a hyperdiverse tropical forest. New Phytologist, 219, 1–13. 10.1111/nph.15206 PMC6001574 29774944

[ece39335-bib-0043] Poorter, L. , Wright, S. J. , Paz, H. , Ackerly, D. D. , Condit, R. , Ibarra‐Manriquez, G. , Harms, K. E. , Licona, J. C. , Martinez‐Ramos, M. , Mazer, S. J. , Muller‐Landau, H. C. , Pena‐Claros, M. , Webb, C. O. , & Wright, I. J. (2008). Are functional traits good predictors of demographic rates? Evidence from five neotropical forests. Ecology, 89, 1908–1920. 18705377 10.1890/07-0207.1

[ece39335-bib-0044] R Core Team . (2020). R: A language and environment for statistical computing. R Foundation for Statistical Computing. https://www.R‐project.org/

[ece39335-bib-0045] Ralph, M. N. , & Christopher, J. W. (2004). Hierarchical partitioning public‐domain software. Biodiversity and Conservation, 13, 659–660.

[ece39335-bib-0046] Reich, B. , Wright, I. J. , Cavender‐Bares, J. , Cavender‐Bares, J. , Craine, J. M. , Oleksyn, J. , Westoby, M. , & Walters, M. B. (2003). The evolution of plant functional variation: Traits, spectra, and strategies. International Journal of Plant Sciences, 164, S143–S164.

[ece39335-bib-0048] Ryan, M. H. (2013). Rmisc: Rmisc: Ryan miscellaneous . R package version 1.5. https://CRAN.R‐project.org/package=Rmisc

[ece39335-bib-0049] Shi, F. S. , Chen, H. F. , & Wu, N. (2008). Effects of warming on carbon and nitrogen contents in subalpine alpine meadows in northwest Sichuan. Bulletin of Botanical Research, 28, 730–736.

[ece39335-bib-0050] Song, T. Q. , Wang, K. L. , Zeng, F. P. , Peng, W. X. , & Du, H. U. (2015). Plant and the environment in karst areas of southwest China. Science Press.

[ece39335-bib-0051] Vile, D. , Shipley, B. , & Garnier, E. (2006). A structural equation model to integrate changes in functional strategies during old‐field succession. Ecology, 87, 504–517. 16637374 10.1890/05-0822

[ece39335-bib-0053] Violle, C. , Navas, M. L. , Vile, D. , Kazakou, E. , Fortuned, C. , Hummel, I. , & Gamier, E. (2007). Let the concept of trait be functional. Oikos, 116, 882–892.

[ece39335-bib-0054] Wang, R. L. , Yu, G. R. , He, N. P. , Wang, Q. F. , Zhao, N. , & Xu, Z. W. (2016). Altitudinal variation in the covariation of stomatal traits with leaf funcyional traits in Changbai Mountain. Acta Ecologica Sinica, 36, 2175–2184.

[ece39335-bib-0055] Wang, S. J. , Li, Y. B. , & Li, R. L. (2003). Karst rocky desertification: Formation background, evolution and comprehensive taming. Quaternary Sciences, 21, 657–666.

[ece39335-bib-0056] Wei, T. Y. , & Viliam S . (2021). R package ‘corrplot’: Visualization of a correlation matrix (version 0.92) . https://github.com/taiyun/corrplot

[ece39335-bib-0057] Wright, I. J. , Reich, P. B. , Westo, Y. M. , Ackerly, D. D. , Baruch, Z. , Bongers, F. , Cavender‐bares, J. , Chapin, T. , Cornelissen, J. H. C. , Diemer, M. , Flexas, J. , Garnier, E. , Groom, P. K. , Gulias, J. , Midgley, J. J. , Navas, M. L. , Ninements, U. , Oleksyn, J. , Osada, N. , … Prior, L. (2004). The worldwide leaf economics spectrum. Nature, 428, 821–827. 15103368 10.1038/nature02403

[ece39335-bib-0058] Xi, X. Q. , Zhao, Y. J. , Liu, Y. G. , Wang, X. , & Gao, X. M. (2011). Variation and correlation of plant functional traits in karst area of central Guizhou Province, China. Journal of Plant Ecology, 35, 1000–1008.

[ece39335-bib-0059] Xu, Y. S. , Huang, H. X. , Shi, Q. R. , Yang, X. D. , Zhou, L. L. , Zhao, Y. T. , Zhang, Q. Q. , & Yan, E. R. (2015). Response of soil water content to change in plant functional traits in evergreen broadleaved forests in eastern Zhejiang Province. Journal of Plant Ecology, 39, 857–866.

[ece39335-bib-0060] Yao, X. Y. , Hu, Y. S. , & Liu, Y. H. (2014). Plant functional traits and functional diversity of different communities in broad‐leaved Korean pine forests in Changbai Mountain. Journal of Northwest A & F University, 42, 77–84.

[ece39335-bib-0061] Yu, L. F. , Zhu, S. Q. , Ye, J. C. , Wei, L. M. , & Chen, Z. R. (2002a). Dynamics of a degraded karst forest in the process of natural recovery. Scientia Silvae Sinicae, 38, 1–7.

[ece39335-bib-0062] Yu, L. F. , Zhu, S. Q. , Ye, J. Z. , Wei, L. M. , & Chen, Z. R. (2002b). Study on human disturbance and degradation and evaluation of karst forest community. Chinese Journal of Applied Ecology, 13, 529–532. 12181889

[ece39335-bib-0063] Zhang, J. Y. (2014). Study on community characteristics of tropical natural coniferous forest‐broad‐leaved forest ecotone in Hainan Island. Chinese Academy of Forestry Sciences.

[ece39335-bib-0064] Zhang, Z. K. , Zheng, X. X. , Lin, H. Z. , Lin, X. , & Huang, L. Q. (2019). Summary of changes in plant functional traits and environmental factors in different recoveryal stages of Island plants. Acta Ecologica Sinica, 39, 3749–3758.

